# Association of Pregnancy Documentation with Radiological Imaging in the Emergency Department

**DOI:** 10.5811/westjem.50731

**Published:** 2026-06-28

**Authors:** Angela G. Campbell, Austin Knies, Paul Musey, Kelli K. Ryckman, Sami Gharbi, Sarah Wiehe

**Affiliations:** * Indiana University, School of Public Health, Department of Applied Health Science, Bloomington, Indiana; † Abt Global, Cambridge, Massachusetts; ‡ Indiana University School of Medicine, Department of Emergency Medicine, Indianapolis, Indiana; § Indiana University, School of Public Health, Department of Epidemiology and Biostatistics, Bloomington, Indiana; ¶ Regenstrief Institute, Indianapolis, Indiana; || Indiana University School of Medicine, Children’s Health Services Research, Indianapolis, Indiana; # Indiana University School of Medicine, Department of Pediatrics, Indianapolis, Indiana

## Abstract

**Introduction:**

Emergency department (ED) encounters are common for pregnant individuals in the United States. Radiological imaging is frequently ordered for diagnostics in the ED but generally avoided during pregnancy. This study examines missed pregnancy documentation and the association of pregnancy documentation with radiological imaging among individuals who have an ED encounter while pregnant.

**Methods:**

We used a retrospective cohort of pregnant individuals who had a live birth in an urban county in Indiana 2022–2023. We pulled all ED encounters occurring between 42–279 days of gestation (N = 4,597) that did not occur within five days of delivery. Our primary outcome measure was pregnancy documentation in the patient’s electronic health record and receipt of radiological imaging (radiograph and computed tomography) of the chest, abdomen or spine. We used descriptive statistics and logistic regressions for the analysis.

**Results:**

Pregnancy was not documented in 273 of the 4,597 encounters (5.9%). A larger percentage of encounters with missed pregnancy documentation occurred with non-Hispanic Black patients (162/273, 59.3%) compared to non-Hispanic White patients (75/273, 27.5%); (odds ratio 1.70, 95% CI, 1.21–2.41) for non-Hispanic Blacks relative to non-Hispanic Whites. Radiological imaging occurred in 147 of the 4,324 encounters with a documented pregnancy (3.4%), compared with 30 of the 273 encounters where the pregnancy was not documented (11.0%). Missed pregnancy documentation was associated with 2.11 times (95%CI, 1.30–3.42) the odds of receiving radiological imaging when adjusted for confounders.

**Conclusion:**

Missed pregnancy documentation occurred in 5.9% of emergency encounters with pregnant patients. While this is uncommon, it is still significantly associated with receipt of radiological imaging. Disparities in odds of pregnancy documentation in the ED may contribute to differences in maternal outcomes and highlights the necessity of ensuring equitable care for all pregnant individuals.

## INTRODUCTION

Compared to other developed countries, the United States has the highest maternal mortality rate.^1^ There are many reasons for this, such as fragmented care,^2^ poor communication between specialists for patients with chronic conditions,^2^ and poor prenatal care.^3^ Pregnant individuals who use the emergency department (ED) are more likely to belong to racial and ethnic minority populations, be publicly insured, be younger, rural, and have less formal education, and have higher health risks.^4^–^7^ Pregnant individuals with pre-existing comorbidities such as hypertension and diabetes are also more likely to use the ED.^8^ In fact, it is estimated that over one-third of pregnant individuals visit the ED at some point in their pregnancy.^4^,^6^

Emergency departments are care settings where the risk for medical error is heightened. Factors associated with this heightened risk include the following: physicians treating unfamiliar patients; crowding and/or understaffing; the need to provide care 24 hours a day; and the urgency of decision-making required for critically ill patients.^9^,^10^ Documenting the pregnancy status of individuals in the ED is crucial, as it informs risk assessment and clinical decision-making. Symptoms such as elevated blood pressure that may be mildly concerning in a non-pregnant patient could indicate serious issues in a pregnant patient.^11^ Treatment pathways and medication choices also change when an individual is pregnant because the physician must consider the health of both the pregnant patient and the fetus.^12^,^13^

Although it may seem intuitive that a pregnant individual would identify themselves as pregnant to clinical staff, or that they could be easily identified visually, this is not always the case. On average, pregnancy is recognized at 5.5 weeks, but 23% of individuals recognize at seven weeks or later.^14^ Later pregnancy recognition is more common among racial and ethnic minorities and for unintended pregnancies.^15^

Radiological imaging, such as radiographs and computed tomography (CT), is frequently used in the ED but is generally avoided during pregnancy unless absolutely necessary.^16^ It has been demonstrated that singular radiographs and CT typically give a dose of radiation that is below the threshold for fetal harm; however, because radiation exposure is cumulative^17^ the standard of practice is to minimize exposure during pregnancy whenever possible.^16^ Imaging is usually reserved for situations where the expected benefits outweigh the risks, and alternatives such as ultrasound or magnetic resonance imaging (MRI) are not available.^18^ While some EDs may have policies related to pregnancy testing before radiological imaging, there are no national guidelines regarding testing,^19^ and some clinical locations may waive testing if the patient states they are not pregnant or reports that the first day of their last menstrual period occurred within 10 days of the date of imaging.^19^

Research has documented a rise in the use of radiological imaging among pregnant individuals^20^,^21^ with evidence that Black pregnant patients are more likely than their White counterparts to receive imaging.^21^ While this trend may partially reflect increased confidence in the safety of imaging during pregnancy, it also raises the possibility that some imaging occurs without knowledge of the pregnancy. In this study we examine how pregnancy documentation is associated with the use of radiological imaging in the ED.

**Population Health Research Capsule**

What do we already know about this issue?

*Emergency department (ED) visits in pregnancy are common. Radiological imaging in the ED is also common but avoided in pregnancy*.

What was the research question?

*Among pregnant patients in the ED, is missed pregnancy documentation associated with more radiological imaging?*

What was the major finding of the study?

*Missed pregnancy documentation was associated with increased odds of radiological imaging (11.0% vs 3.4%; aOR 2.11, 95% CI 1.30–3.42)*.

How does this improve population health?

*Improving pregnancy documentation/screening in the ED can reduce unnecessary radiation, guide safer medications/triage, and address inequities in patient care*.

## METHODS

### Study Design and Data Source

We constructed a retrospective cohort study of pregnant individuals with a live birth in any hospital located within an urban county in Indiana that was part of a single, large health system between January 2022–July 2023 using electronic health records (EHR) data. The primary outcomes of interest were missed pregnancy documentation (defined below) and radiological imaging of the abdomen, chest, or spine. Our final sample included all ED encounters occurring between 42 days (six weeks) and 279 days (40 weeks minus one day) of gestation within a pregnancy period for pregnant patients in this retrospective cohort. The data used in this sample were first sourced from encounter data for the retrospective cohort. Using the pregnant patient’s unique health system identification number, we merged additional data from person files, diagnosis code files, orders files, and events files to the encounter data to obtain additional patient and encounter characteristics for the analysis.

We further limited pregnancy-specific encounters for pregnant patients in our cohort to those identified as an ED encounter and to those encounters with at least one diagnosis code. We removed observations that occurred on or within five days of the delivery date and that were duplicates for pregnant individuals with more than one live birth in the same pregnancy. If there was *International Classification of Diseases, 10^th^ Rev* (*ICD-10*) code documentation of a delivery prior to 40 weeks (eg, delivery at 31 weeks), then we adjusted gestational age to reflect this and used this information to limit encounters. In this example, an infant born at 31 weeks would have a 217-day window for encounters, and only those encounters between 42 days (six weeks) and 217 days (31 weeks) that did not occur within five days of the delivery date would be included. All *ICD-10* codes used in this study are listed in Appendix A.

Pregnancy is typically not detectable until the fourth week of pregnancy,^22^ and gestational age estimates can be off by as much as 14 days if an ultrasound was not obtained in the first trimester.^23^ Therefore, in an effort to conduct conservative analyses, we only included pregnancies that were at least six weeks along and, thus, detectable via a urine test for pregnancy. Emergency encounters that occurred through the end of the pregnancy were included to get a more accurate picture of the frequency of missed pregnancy documentation throughout the pregnancy. Thirteen observations lacked documentation of race/ethnicity. Because we considered this to be not large enough to justify making it a separate category or to justify more advanced data imputation procedures, these observations were dropped. Our data-cleaning process resulted in a study sample of 4,597 ED encounters for pregnant patients who had a live birth within a large health network in an urban Indiana county between January 2022–July 2023. The institutional review board (IRB) agreement allowed for sampling of births occurring within one health system in a single county. However, all encounter data within the health system was examined so that all emergency encounters, including those that occurred outside the county of birth were included, provided that the facility offering emergency care was part of the health system of birth.

Because pulling EHR data for a study differs from manual chart abstraction, we used data scientists and coders to pull the study sample and define an algorithm to identify cases rather than training abstractors and using abstraction forms. The data scientists were not blinded to the hypothesis as their expertise was used to help us identify the data fields we needed to define our variables. Their performance was not monitored in a formal manner, and there was no inter-rater reliability discussed or tested because one person was responsible for writing each portion of the code, although the contents of the code/algorithm were discussed at length. There are, however, some best practices related to manual chart abstraction that we adhered to.^24^ The variables in this study are clearly defined, the medical record database is identified as records pulled from a large health system in the Midwest, the sampling method was described, and IRB approval was obtained. There was very little missing data, with only 13 observations being dropped due to lack of race/ethnicity data; this comprised just 0.28% of the sample and was determined to be acceptable.

### Variables

#### Radiological Imaging

In this study, the primary outcome of interest was whether radiological imaging of the abdomen, chest or spine was performed on a pregnant patient during an ED encounter that fell between 42-279 days gestation and did not occur within five days of delivery. Using data from medical orders and events files merged to each encounter, we identified whether radiological imaging (either radiograph or CT) was performed during the encounter. If there was documentation in the order status fields that the ordered radiograph or CT was canceled or terminated without results, then that scan was excluded. We could not determine the reason for the cancelation from the abstracted data.

#### Missed Pregnancy Documentation

The treatment variable in this study was whether the ongoing pregnancy was documented during the encounter. Due to the methods we used to filter the sample, all pregnant individuals in the sample were pregnant during each ED encounter. The flag for a missed pregnancy documentation was meant to identify whether the pregnancy had not been documented in the EHR by the medical staff during the ED encounter. We assigned a missed pregnancy-documentation flag to the encounter if the following criteria held: 1) no prenatal care or obstetric diagnosis codes were issued for the encounter (defined in Appendix A); 2) pregnancy was not acknowledged in the descriptive variables in the EHR data (this included a free-text note field attached to the encounter record and a free-text reason for visit variable); and 3) no pregnancy test was given during the encounter. When the pregnancy was documented, 96.6% of the time it was done via *ICD-10* code. A full breakdown of the location of pregnancy documentation in the patient’s record is available in Appendix B.

#### Control Variables

Using data matched to the pregnant patient, we extracted the race/ethnicity (non-Hispanic White, non-Hispanic Black, Hispanic and Asian/other), insurance status at delivery (commercial/private, Medicaid/public, uninsured/self-pay/other), and age (in years) of the pregnant individual at the time of encounter. We also extracted encounter characteristics in the data such as time of day of encounter (grouped into four-hour increments), and month, year and location of encounter. The location variable was grouped into encounters at county hospital A, county hospital B, and encounters that occurred outside the county. The number of weeks gestation at the time of the encounter was coded continuously, with a squared term in the analytic models to account for non-linearity. Finally, we used the *ICD-10* diagnosis codes associated with each encounter to construct flags if the encounters were trauma-related or had diagnoses of pain in the abdomen, chest or back (*ICD-10* codes listed in Appendix A).

### Statistical Methods

We examined descriptives statistics followed by bivariate logistic and multivariate logistic regressions, and we used Stata 18 (StataCorp, LLC, College Station, TX) for all analyses. These data were at the encounter level, and some pregnant individuals may have had multiple ED encounters during their pregnancy. To account for repeated measures for an individual, all models were estimated with a specification to cluster the error term by the pregnant patient’s ID number. This study follows the Strengthening the Reporting of Observational Studies in Epidemiology cohort reporting guideline. The primary outcomes are pregnancy documentation and radiological imaging. The secondary outcome is race/ethnicity as it is associated with pregnancy documentation and radiological imaging.

## RESULTS

Within this sample of 4,597 ED encounters, 5.9% had a missed pregnancy documentation, and 3.9% of the encounters included radiological imaging of the abdomen, chest, or spine (Table 1). Minoritized racial/ethnic groups made up 61.2% of the sample, and 80.5% of pregnant individuals in this sample were insured by Medicaid or uninsured at the time of delivery. Most of the patients in the sample (86.0%) gave birth between the ages of 18–34. Trauma *ICD-10* codes were present in 5.2% of encounters, and *ICD-10* codes indicating pain were present in 26.5% of encounters. The average number of weeks gestation at the time of the encounters was 25 (SD 10.3).

More than three quarters (77.7%) of missed pregnancy documentation occurred in the first or second trimester (Table 2). Radiological imaging occurred in 147 of the 4,324 encounters with a documented pregnancy (3.4%), and 30 of the 273 encounters where the pregnancy was not documented (11.0%).

In univariate logistic regression models, non-Hispanic Black pregnant patients had 1.70 times the odds of not having their pregnancy documented (95% CI, 1.21–2.41) (Table 3) compared to non-Hispanic White pregnant patients. Pregnant individuals with a trauma *ICD-10* code did not have significantly different odds of having a missed pregnancy documentation relative to pregnant individuals without a trauma *ICD-10* code. Receipt of a pain-related *ICD-10* code was associated with 0.16 times reduced odds (an 84% reduction) of a missed pregnancy documentation (95% CI, 0.10–0.25)]. Receipt of radiological imaging of the abdomen, chest or spine was not significantly associated with race/ethnicity, age at delivery, or type of insurance in the bivariate analyses. Receipt of a trauma *ICD-10* code was associated with 2.54 times the odds of receiving radiological imaging (95% CI, 1.62–3.98). Receipt of a pain-related *ICD-10* code was significantly associated with 1.46 times the odds of receiving radiological imaging (95% CI, 1.06,2.02).

Missed pregnancy documentation was associated with 1.91 times the odds of receiving radiological imaging of the abdomen chest or spine (95% CI, 1.24–2.98) in the model that was not adjusted for sociodemographic or clinical factors (Table 4). In the fully adjusted model, missed pregnancy documentation was associated with 2.11 times the odds of receiving radiological imaging (95% CI, 1.30–3.42). Receipt of a trauma-related *ICD-10* code was associated with 2.20 times the odds of radiological imaging the fully adjusted model 95% CI, 1.39–3.50). Receipt of a pain-related *ICD-10* was associated with 1.63 times the odds of radiological imaging (95% CI, 1.15, 2.30).

## DISCUSSION

In this study we found that pregnancy was not documented in 5.9% of ED encounters. Missed documentation was more likely to occur among patients who were non-Hispanic Black, and the lack of documentation was significantly associated with receipt of radiological imaging (radiograph or CT). Given that radiological imaging is typically avoided during pregnancy unless necessary,^16^ these findings underscore the critical role of accurate documentation in ensuring optimal clinical decision-making and safeguarding maternal and fetal health.

It is important to acknowledge that some degree of medical error is inevitable in the ED environment, where crowding, understaffing, and limited access to patient histories pose significant challenges.^10^,^25^ Further, if a patient is in critical condition, the necessity of diagnostic imaging may outweigh the radiation risks posed to the pregnant individual or fetus. The positive association of trauma *ICD-10* codes with missed pregnancy documentation, suggests that this may apply to a portion of the sample. However, only 25 of the 273 (9.2%) encounters with a missed pregnancy documentation had a trauma code. Additionally, the results demonstrate that even when controlling for trauma and pain, the likelihood of receiving imaging is not distributed equally between pregnant patients whose pregnancy is documented in their record and those whose pregnancy is not documented. This suggests that some of these patients might have been evaluated using alternative modalities such as MRI had their pregnancy status been known.

Missed pregnancy documentation was disproportionately common among non-Hispanic Black compared to non-Hispanic White individuals. This aligns with prior qualitative research indicating that non-Hispanic Black individuals experience poor communication with clinicians when receiving medical care.^26^ Unlike in previous studies, we did not find that race and ethnicity were significantly associated with receipt of imaging^21^ in the bivariate or multivariate models; however, this does not make the relationship of race and ethnicity with a missed pregnancy documentation less concerning. Pregnancy documentation may influence how the individual is triaged (eg, high blood pressure could be an indicator of preeclampsia in pregnant individuals) and what medications are prescribed.^27^ Non-Hispanic Black patients who are pregnant already face the highest rates of maternal morbidity and mortality in the U.S. relative to all other racial and ethnic groups,^28^ and are more likely to receive care in the ED relative to non-Hispanic White individuals^5^; therefore, minimizing risk to this population is a priority. Non-Hispanic Black individuals also tend to recognize their pregnancies later than non-Hispanic Whites,^14^ highlighting a potential need for more systematic pregnancy testing protocols in the ED to account for cases where the individual may not yet be aware of their pregnancy. Understanding the reasons behind the lower rates of pregnancy documentation in this population could inform targeted interventions to improve care.

## LIMITATIONS

This study is not without limitations. First, Current Procedural Terminology codes were not available for this study, which could have impacted our documentation measure. Second, while most documentation was done using *ICD-10* code, these codes were likely not entered directly by the clinician providing care to the pregnant individual. Physicians, especially those in large practices, often document patient information in structured notes in the patient’s record,^29^,^30^ and these notes are later turned into diagnosis codes by trained coders.^31^ Some documentation of pregnancy could have been “lost in translation,” if it was not deemed to be a billable *ICD-10* code diagnosis or it was recorded in the patient’s chart outside the free-text fields included in this study. Therefore, absence of pregnancy documentation in the patient’s record does not necessarily indicate that the pregnancy was not verbally discussed. Third, some patients may have chosen not to disclose their pregnancy status to their clinician due to privacy concerns. Further qualitative research is needed to explore whether such concerns contribute to missed documentation and affect care.

Finally, our study population consisted of pregnant individuals who delivered within the same health system in one geographic county during a defined period. As a result, the sample does not represent national trends or even all ED encounters involving pregnant patients at these facilities. Pregnant patients who received care at these EDs but did not deliver within the sample health system and sample county were not included, and we cannot assess how this might have influenced our estimates of documentation. Indeed, the rarity of studies on missed pregnancy documentation in the ED may reflect the difficulty of identifying undocumented pregnancies, as large public datasets such as the National Hospital Ambulatory Medical Care Survey lack the means to identify pregnant patients who had no pregnancy recorded during their ED visit.

## CONCLUSION

Over one third of pregnancies in the U.S. have at least one emergency department encounter,^4^,^6^ and understanding how to provide optimal care to pregnant patients in need of emergency services is important for maternal health. This study leveraged rich electronic health records data to examine the impact of pregnancy documentation during an ED encounter on the use of radiological imaging as a clinical outcome. Pregnancy was not documented in 5.9% of ED encounters, with missed documentation disproportionately affecting non-Hispanic Black individuals. The absence of pregnancy documentation was significantly associated with increased use of radiological imaging, even after accounting for trauma-related cases. While documentation errors in the ED are serious,^32^ they may be due to systemic issues that require interventions at multiple levels^10^,^32^,^33^ rather than simple interventions at the clinician level. Interventions to improve pregnancy documentation—such as standardized screening protocols or improved clinician support—may be essential steps toward advancing care quality and equity.

## Supplementary Information





## Figures and Tables

**Table 1:** Descriptives of emergency department encounters that occurred during pregnancy (N = 4,597) in a group of patients who gave birth in a single, urban county in Indiana, 2022–2023.

Outcome variables	N or Mean	% or SD
Pregnancy documentation at encounter		
Documented	4,324	94.1%
Not documented	273	5.9%
Receipt of radiological imaging for abdomen chest, or spine, at ED encounter		
Yes	177	3.9%
No	4,420	96.2%
Sociodemographic variables	N or Mean	% or SD
Pregnant individual’s race		
Non-Hispanic White	1,783	38.8%
Non-Hispanic Black	1,901	41.4%
Hispanic	777	16.9%
Asian/Other	136	3.0%
Age at time of delivery		
<18	97	2.1%
18–25	2,051	44.6%
26–34	1,902	41.4%
≥35	547	11.9%
Insurance at delivery		
Commercial/Private	896	19.5%
Medicaid/Public	3,617	78.7%
Uninsured/Self-Pay/Other	84	1.8%
Clinical variables	N or Mean	% or SD
Trauma *ICD-10* diagnosis		
Yes	238	5.2%
No	4,359	94.8%
Pain *ICD-10* diagnosis		
Yes	1,219	26.5%
No	3,378	73.5%
Number of weeks gestation at time of encounter	25	(10.3)
Time and location variables	N or Mean	% or SD
Location of emergency encounter		
Location 1	1,038	22.6%
Location 2	3,016	65.6%
Location 3	543	11.8%
Year of ED encounter		
2021	428	9.3%
2022	3,059	66.5%
2023	1,110	24.2%

*ED*, emergency department; *ICD-10, International Classification of Diseases, 10^th^ Revision*; *SD*, standard deviation.

**Table 2:** Cross-tabulation of study variables by pregnancy documentation status for emergency department encounters that occurred while pregnant (N = 4,597) in a retrospective cohort of individuals who gave birth in a single urban county in Indiana, 2022–2023.

	Pregnancy was documented (N = 4,324)	Pregnancy was NOT documented (N = 273)
Receipt of radiological imaging for abdomen chest, or spine, at ED encounter	N (%)	N (%)
Yes	147 (3.4%)	30 (11.0%)
No	4,177 (96.6%)	243 (89.0%)
Sociodemographic variables*		
Pregnant individual’s race		
Non-Hispanic White	1,708 (39.5%)	75 (27.5%)
Non-Hispanic Black	1,739 (40.2%)	162 (59.3%)
Asian/Hispanic/Other	877 (20.3%)	36 (13.2%)
Age at Delivery		
<18–25	2,006 (46.4%)	142 (52.0%)
26–34	1,805 (41.7%)	97 (35.5%)
≥35	513 (11.9%)	34 (12.5%)
Insurance at delivery		
Commercial/Private	854 (19.8%)	42 (15.4%)
Medicaid/Public	3,396 (78.5%)	221 (81.0%)
Uninsured/Self-Pay/Other	74 (1.7%)	10 (3.7%)
Clinical variables		
Trauma *ICD-10* diagnosis		
Yes	213 (4.9%)	25 (9.2%)
No	4,111 (95.1%)	248 (90.8%)
Pain *ICD-10* diagnosis		
Yes	1,195 (27.6%)	24 (8.8%)
No	3,129 (72.4%)	249 (91.2%)
Trimester of encounter		
First	885 (20.5%)	86 (31.5%)
Second	1,209 (28.0%)	126 (46.2%)
Third	2,230 (51.6%)	61 (22.3%)

* Some categories were combined to meet reporting requirements.

*ED*, emergency department; *ICD-10, International Classification of Diseases, 10^th^ Revision*.

**Table 3:** Bivariate logistic regressions examining the relationship of sociodemographic and clinical variables with pregnancy documentation and radiological imaging at emergency department encounters that occurred while pregnant (N = 4,597) in a retrospective cohort of individuals who gave birth in a single urban county in Indiana 2022–2023.

	Missed pregnancy documentation		Receipt of radiological imaging for abdomen, chest or spine	
	
	OR	95% CI	OR	95% CI
Pregnant Individual’s Race				
Non-Hispanic White	ref		ref	
Non-Hispanic Black	1.70	(1.21, 2.41)	0.90	(0.62, 1.31)
Hispanic	1.07	(0.66, 1.74)	0.92	(0.56, 1.51)
Asian/Other	0.68	(0.26, 1.79)	1.55	(0.71, 3.39)
Age at time of delivery				
<18	ref		ref	
18–25	1.14	(0.33, 3.90)	1.07	(0.37, 3.03)
26–34	0.94	(0.28, 3.20)	1.09	(0.39, 3.10)
≥35	1.03	(0.30, 3.58)	1.50	(0.50, 4.56)
Insurance at delivery				
Commercial/Private	ref		ref	
Medicaid/Public	1.30	(0.89, 1.88)	0.73	(0.49, 1.08)
Uninsured/Self-Pay/Other	2.18	(0.93, 5.08)	0.20	(0.03, 1.49)
Trauma *ICD-10* diagnosis				
Yes	1.12	(0.69, 1.83)	2.54	(1.62, 3.98)
No	ref		ref	
Pain *ICD-10* diagnosis				
Yes	0.16	(0.10, 0.25)	1.46	(1.06, 2.02)
No	ref		ref	
Number of weeks gestation at time of encounter	1.29	(1.18, 1.40)	1.19	(1.08, 1.32)
Weeks gestation squared	0.995	(0.993, 0.997)	0.996	(0.994, 0.999)

* Controls for time of day, month, year, and location of encounter were included in all bivariate regressions.

*ICD-10, International Classification of Diseases, 10^th^ Revision; OR*, odds ratio.

**Table 4:** Logistic regressions examining the relationship between missed pregnancy documentation in an emergency department encounter with odds of receipt of radiological imaging that occurred while pregnant (N = 4,597) in a retrospective cohort of individuals who gave birth in a single urban county in Indiana 2022–2023.

	Model 1		Model 2	
	
	OR	95% CI	OR	95% CI
Pregnancy documentation at encounter				
Documented	ref			
Not Documented	1.91	(1.24, 2.98)	2.11	(1.30, 3.42)
Pregnant Individual’s Race				
Non-Hispanic White			ref	
Non-Hispanic Black			0.90	(0.60, 1.34)
Hispanic			1.02	(0.62, 1.68)
Asian/Other			1.65	(0.73, 3.73)
Age at time of delivery				
<18			ref	
18–25			1.15	(0.42, 3.19)
26–34			1.23	(0.44, 3.41)
≥35			1.57	(0.53, 4.66)
Insurance at Delivery				
Commercial/Private			ref	
Medicaid/Public			0.78	(0.51, 1.19)
Uninsured/Self-Pay/Other			0.23	(0.03, 1.67)
Trauma *ICD-10* diagnosis				
Yes			2.20	(1.39, 3.50)
No			ref	
Pain *ICD-10* diagnosis				
Yes			1.63	(1.15, 2.30)
No			ref	
Number of weeks gestation at time of encounter			1.16	(1.04, 1.29)
Weeks gestation squared			0.997	(0.995, 0.999)

* Controls for time of day, month, year, and location of encounter were included in all regressions.

*ED*, emergency department; *ICD-10, International Classification of Diseases, 10^th^ Revision; OR*, odds ratio.
