# Supplementary material for: Association of Pregnancy Documentation with Radiological Imaging in the Emergency Department
Source: West J Emerg Med. 2026 Jun 28;27(4):985–93. doi: 10.5811/westjem.50731 (PMC13436638; doi:10.5811/westjem.50731)
Supplement: Supplementary file 1 [file wjem-27-985-s001.docx]

**Appendix B.** Venn Diagram of the Location of Pregnancy Documentation in the Patient’s Electronic Health Record for encounters that occurred in an emergency department while pregnant in a retrospective cohort of individuals who gave birth in an urban county in Indiana 2022-2023
