# Supplementary material for: Association of Pregnancy Documentation with Radiological Imaging in the Emergency Department
Source: West J Emerg Med. 2026 Jun 28;27(4):985–93. doi: 10.5811/westjem.50731 (PMC13436638; doi:10.5811/westjem.50731)
Supplement: Supplementary file 2 [file wjem-27-985-s002.docx]

Appendix A. ICD-10 used for data extraction and analysis in a retrospective cohort study of emergency department encounters among individuals who gave birth in an urban hospital in Indiana 2022-2023

| **Diagnosis** | **Search Term/Code Used in EHR** |
| --- | --- |
| **Obstetrics** |  |
| Weeks of gestation | Z3A* |
| Prenatal Care | Z363*, Z34*, |
| Encounter for a pregnancy test and/or childbirth/childcare instruction | Z32* |
| Pregnancy state, incidental | Z33.1 |
| Supervision of pregnancy with other poor reproductive or obstetric history | O09.2 |
| Supervision of pregnancy with insufficient antenatal care | O09.3 |
| Supervision of high-risk pregnancy due to social problems | O09.7 |
| Supervision of high-risk pregnancy other | O09.1*, O09.4*. O09.5*, O09.6*. O09.8*, O09.9*, O09.A* |
| Pre-existing hypertension complicating pregnancy, childbirth and the puerperium | O10* |
| Pre-existing hypertension with pre-eclampsia | O11* |
| Gestational [pregnancy-induced] edema and proteinuria without hypertension | O12* |
| Gestational [pregnancy-induced] hypertension without significant proteinuria | O13* |
| Pre-eclampsia | O14* |
| Eclampsia | O15* |
| Unspecified maternal hypertension | O16* |
| Supervision of high-risk pregnancy | O09*, O20*, O21*, O22*, O23*, O25*, O26* , O29* |
| Gestational Diabetes | O24* |
| Multiple Gestation | O30*, O31* |
| Premature Rupture of Membranes | O42* |
| Placental Disorders | O43* |
| Placenta Previa | O44* |
| Premature Separation of Placenta | O45* |
| Antepartum Hemorrhage (not elsewhere classified) | O46* |
| Preterm Labor | O60* |
| Failed induction of labor | O61* |
| Labor Complications Other | O64*, O65*, O66*, O67*, O68*, O69*, O71*, O73*, O74*, O75*, O76*, O77* (Does not include O70 - perineal laceration trauma) |
| Postpartum Hemorrhage | O72* |
| Encounter for delivery | O80*, O82* |
| Complication related to the puerperium | O85*, O86*, O87*, O88*, O89*, O90*,  (Does not include complications related to breastfeeding O91*, O92*) |
| Abuse during Pregnancy | O9A* |
| **Trauma** |  |
| Injuries to the neck | S10*-S19* |
| Injuries to the thorax | S20*-S29* |
| Injuries to the abdomen, lower back, lumbar spine, pelvis and external genitals | S30*-S39* |
| Injuries to the shoulder and upper arm | S40*-S49* |
| Injuries to the elbow and forearm | S50*-S59* |
| Injuries to the wrist, hand and fingers | S60*-S69* |
| Injuries to the hip and thigh | S70*-S79* |
| Injuries to the knee and lower leg | S80*-S89* |
| Injuries to the ankle and foot | S90*-S99* |
| Injuries involving multiple body regions | T07* |
| Injuries to unspecified body region | T14* |
| Effects of foreign body entering through orifice | T15*-T19* |
| Burns and corrosions | T20*-T32* |
| Frostbite | T33*-T34* |
| Poisoning by drugs, medicaments and biological substances | T36*-T50* |
| Toxic effects of substances chiefly nonmedicinal as to source | T51*-T65* |
| Other and unspecified effects of external causes | T66*-T78* |
| Certain early complications of trauma | T79* |
| Transport accidents | V01*-V99* |
| Falls | W00*-W19* |
| Exposure to inanimate mechanical forces | W20*-W49* |
| Exposure to animate mechanical forces | W50*-W64* |
| Accidental drowning and submersion | W65*-W74* |
| Exposure to electric current, radiation and extreme temperature/pressure | W85*-W99* |
| Exposure to smoke, fire and flames | X00*-X09* |
| Contact with heat and hot substances | X10*-X19* |
| Overexertion, travel and privation | X50*-X57* |
| Intentional self-harm | X60*-X84* |
| Assault | X92*-Y09* |
| Event of undetermined intent | Y10*-Y34* |
| Legal intervention, operations of war and terrorism | Y35*-Y38* |
| **Pain** |  |
| Abdominal Pain | R10* |
| Back Pain | M54.5*, M54.9 |
| Chest Pain | R07.1, R07.2, R07.4, R07.8*, R07.9 |
